# Complete Eradication of Bleeding Duodenal Varices with Endoscopic Polydocanol Sclerotherapy

**DOI:** 10.5005/jp-journals-10018-1194

**Published:** 2016-12-01

**Authors:** Tuncer Temel, Abdülvahhap Aktas, Safak Meric Ozgenel, Aysegül Özakyol

**Affiliations:** 1 Department of Gastroenterology, Eskisehir Osmangazi University, Eskisehir, Turkey; 2 Department of Internal Medicine, Eskisehir Osmangazi University, Eskisehir, Turkey

**Keywords:** Cirrhosis, Duodenal varices, Ectopic variceal bleeding, Portal hypertension, Sclerotheraphy.

## Abstract

Bleeding from duodenal varices is a rare complication of portal hypertension, occurring in only 0.4% of these patients and is often life-threatening because of the difficulty in diagnosis and treatment. Treatment options include surgical procedures and endoscopic and endovascular treatments. A 48-year-old female cirrhotic patient was admitted to our clinic with upper gastrointestinal (GI) tract bleeding. Endoscopic examination revealed nonbleeding Lm, Cb, RC (+), F3-F3-F2 esophageal and nodular-bleeding-oozing duodenal varices. Esophageal varices were eradicated with band ligation at two sessions. After one session of 2% polydocanol sclerotheraphy, no signs of bleeding were determined. Complete eradication was achieved after five sessions and 1 year apart from the initial treatment duodenal varices were eradicated. Although duodenal varices are rare, they are frequently fatal. There are limited data regarding optimal treatment. Successful treatment depends both on patient factors (hepatic synthetic function, comorbidities, size/location of the varices) and center expertise. Long-term eradication is variable and may depend on the cause and extensiveness of the ectopic varices.

**How to cite this article:**

Temel T, Aktas A, Ozgenel SM, Özakyol A. Complete Eradication of Bleeding Duodenal Varices with Endoscopic Polydocanol Sclerotherapy. Euroasian J Hepato-Gastroenterol 2016;6(2):176-178.

## INTRODUCTION

Bleeding from duodenal varices is a rare complication of portal hypertension, occurring in only 0.4% of these patients and is often life-threatening because of the difficulty in diagnosis and treatment.^1^ Mortality rate is approximately 40%.^2^ Optimal treatment mode is controversial since data about duodenal variceal bleeding at the literature is limited. Treatment options include surgical procedures and endoscopic and endovascular treatments.^3^ We report an upper gastrointestinal (GI) tract bleeding at a cirrhotic patient with missdiagnosed duodenal varices even after five upper GI tract endoscopic examinations.

## CASE REPORT

**Fig. 1:**
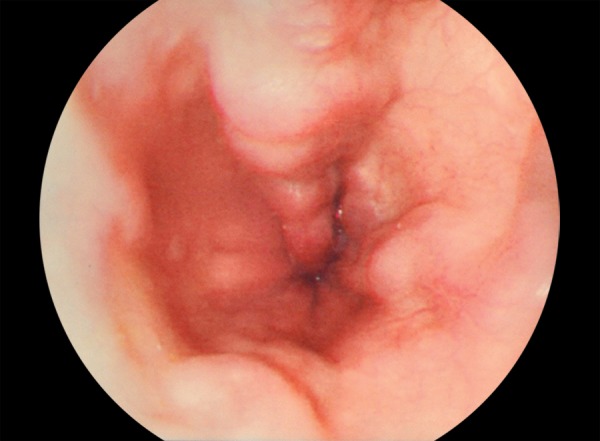
Varices in patient with liver cirrhosis

**Fig. 2:**
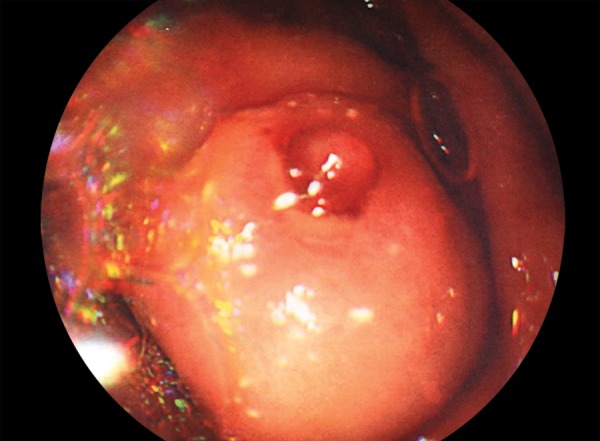
Varices in patient with liver cirrhosis

**Fig. 3:**
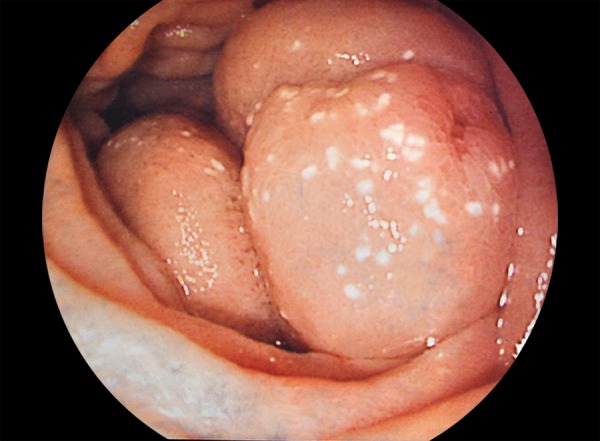
Therapeutic control of variceal bleeding

**Fig. 4:**
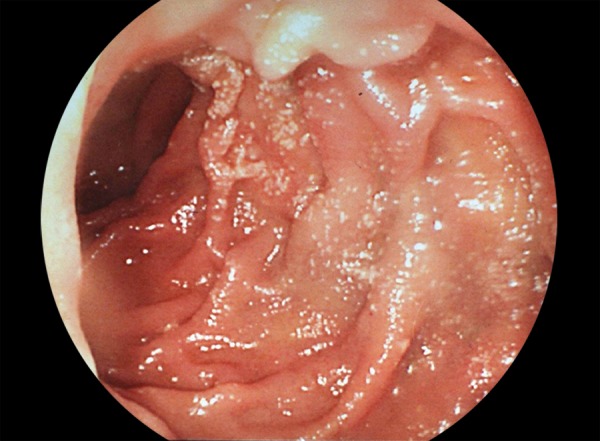
Therapeutic control of variceal bleeding

A 48-year-old female cirrhotic patient admitted to our clinic with upper GI tract bleeding. Prior to admission, five upper GI tract endoscopic examinations were performed with missdiagnosis of duodenal varices. Laboratory findings were as follows: Hemoglobin 6.8 mg/dL, hematocrit 20.4%, white blood cell count 4000/μL, platelets 42000/μL, total/direct bilirubin 1.36 mg/dL, serum albumin 2.8 mg/dL, aspartate aminotransferase (AST) 28 IU/mL, alanine aminotransferase 13 IU/mL, and *international normalized ratio (INR)* 1.38 (reference 0.8–1.2). Neither ascites nor encephalopathy was observed. Child–Pugh’s classification was graded as stage B. Endoscopic examination revealed nonbleeding Lm, Cb, RC(+), F3-F3-F2 esophageal and nodular-bleeding-oozing duodenal varices (Figs 1 and 2). Esophageal varices were eradicated with band ligation at two sessions. After one session of 2% polydocanol sclerotheraphy, no signs of bleeding were determined (Fig. 3), and complete eradication was achieved after five sessions (Fig. 4), and 1 year apart from the initial treatment duodenal varices were eradicated.

## DISCUSSION

The pathologic variceal sites commonly seen are gastroesophageal varices (esophageal varices and cardiofundic varices). Ectopic varices which represent 2 to 5% of GI tract variceal bleeding are dilated splanchnic (mesoportal) veins/varicosities, and/or dilated portosystemic collaterals commonly occur secondary to portal hypertension along the entire GI tract outside the common pathologic variceal sites.^4^ Although distal varices can be determined, duodenal varices are typically located in the 1st or 2nd portions of the duodenum and are commonly identified by upper endoscopy, computed tomography (CT), or mesenteric angiography.^5^ Treatment options in duodenal variceal bleeding include endoscopic procedures (endoscopic varix band ligation (EVL), sclerotherapy, clipping), interventional radiological procedures (TIPS), percutaneous transhepatic obliteration (PTO), transileocolic vein obliteration (TIO), balloon-occluded retrograde transvenous obliteration (BRTO), and surgery (variceal ligation, duodenal resection, and extrahepatic portosystemic shunt creation).^6,7^ Unfortunately, the case numbers of ectopic varices in the literature is small, and no definite conclusion can be made as to which is the ideal way to manage these varices. Endoscopic therapies include mechanical therapies (band ligation) and injection therapies (sclerotherapy with sclerosants or tissue adhesives).^3,8^ There is theoretically an increased risk of complication of banding and sclerotherapy in the duodenum because of the thinness of the wall of this organ. Although the efficacy of endoscopic band ligation of esophageal varices is well established, its use in duodenal variceal bleeding is limited to only case reports. An extensive literature review from 1995 to the present found only 19 previously reported cases of duodenal varices treated with EVL.^9^ The success of duodenal EVL is significant only in 3 of 19 patients (15.8%) with high amount of rebleeding after treatment. No death occurred related with the complications of the procedure or rebleeding. Various sclerosants, such as asethanolamine oleate, sodium morrhuate, absolute alcohol, polydocanol, N-butyl-2-cyanoacrylate, and thrombin have been used successfully in patients with ectopic variceal bleeding in primary endoscopic hemostasis or secondary therapy following failure of other endoscopic approaches.^10^

Although duodenal varices are rare, they are frequently fatal. There are limited data regarding optimal treatment. Successful treatment depends both on patient factors (hepatic synthetic function, comorbidities, and size/location of the varices) and center expertise. Long-term eradication is variable and may depend on the cause and extensiveness of the ectopic varices. In our case, we were successful in achieving hemostasis by using endoscopic injection sclerotherapy with 2% polydocanol, which resulted with the complete eradication of duodenal varices after five sessions. One year apart from the initial treatment duodenal varices were eradicated. Mis diagnosis in five prior upper GI tract endoscopic examinations suggests that endoscopy must be performed by an expert clinician, and the clinicians must consider that the varices can occur at places apart from esophagus and stomach, like distal parts of the duodenum. Endoscopic injection sclerotherapy with polydocanol may be an effective therapeutic option for the control and eradication of ruptured duodenal variceal bleeding.

## References

[1] Hashimoto R, Sofue K, Takeuchi Y, Shibamoto K, Arai Y (2013). Successful balloon-occluded retrograde transvenous obliteration for bleeding duodenal varices using cyanoacrylate.. World J Gastroenterol.

[2] Norton ID, Andrews JC, Kamath PS (1998). Management of ectopic varices.. Hepatology.

[3] Kakizaki S, Toyoda M, Ichikawa T, Sato K, Takaqi H, Arai H, Sohara N, Iizuka H, Onozato Y, Mori M (2010). Clinical characteristics and treatment for patients presenting with bleeding duodenal varices.. Dig Endosc.

[4] Saad W, Lippert A, Nael ES, Caldwell S (2013). Ectopic varices: anatomical classification, hemodynamic classification, and hemodynamic-based management.. Tech Vasc Interv Radiol.

[5] Takamatsu T, Ootake H, Uehara T, Shindou Y, Ikeya T, Toukai K, Ikeda M, Ushimaru S, Asano T, Matsumoto S, Iwaki T (2011). A case of ruptured duodenal varices treated successfully by endoscopic injection sclera-therapy under radiographic guidance with a mixture of N-butyl-2-cyanoacrylate-lipiodol.. Jichi Med Univ J.

[6] Kinzel J, Pichetshote N, Dredar S, Aslanian H, Nagar A (2014). Bleeding from a duodenal varix: a unique case of variceal hemostasis achieved using EUS-guided placement of an embolization coil and cyanoacrylate.. J Clin Gastroenterol.

[7] Kang HY, Lee WK, Kim YH, Kwon BW, Kang MS, Kim SB, Song IH (2011). Ruptured duodenal varices arising from the main portal vein successfully treated with endoscopic injection sclerotherapy: a case report.. Korean J Hepatol.

[8] Soga K, Tomikashi K, Fukumoto K, Miyawaki K, Okuda K, Konishi H, Yagi N, Wakabayashi N, Kokura S, Naito Y, et al. (2010). Successful endoscopic hemostasis for ruptured duodenal varices after balloon-occluded retrograde transvenous obliteration.. Dig Endosc.

[9] Gunnerson AC, Diehl DL, Nguyen VN, Shellenberger MJ, Blansfield J (2012). Endoscopic duodenal variceal ligation: a series of 4 cases and review of the literature.. Gastrointestinal Endosc.

[10] Tan A, Kenneth JK, Zachary MB, Amy W, Sarah AR (2008). Duodenal variceal bleeding successfully treated with transjugular intrahepatic portosystemic shunt: a case report and review of the literature.. Turkish J Gastroenterol.

